# PASSIOMA: Exploring Expressed Sequence Tags during Flower Development in *Passiflora* spp.

**DOI:** 10.1155/2012/510549

**Published:** 2012-03-22

**Authors:** Lucas Cutri, Marcelo Carnier Dornelas

**Affiliations:** Departamento de Biologia Vegetal, Instituto de Biologia, Universidade Estadual de Campinas, Rua Monteiro Lobato 255, 13083-860 Campinas, SP, Brazil

## Abstract

The genus *Passiflora* provides a remarkable example of floral complexity and diversity. The extreme variation of *Passiflora* flower morphologies allowed a wide range of interactions with pollinators to evolve. We used the analysis of expressed sequence tags (ESTs) as an approach for the characterization of genes expressed during *Passiflora* reproductive development. Analyzing the *Passiflora* floral EST database (named PASSIOMA), we found sequences showing significant sequence similarity to genes known to be involved in reproductive development such as MADS-box genes. Some of these sequences were studied using RT-PCR and *in situ* hybridization confirming their expression during *Passiflora* flower development. The detection of these novel sequences can contribute to the development of EST-based markers for important agronomic traits as well as to the establishment of genomic tools to study the naturally occurring floral diversity among *Passiflora* species.

## 1. Introduction

**Figure 1:**
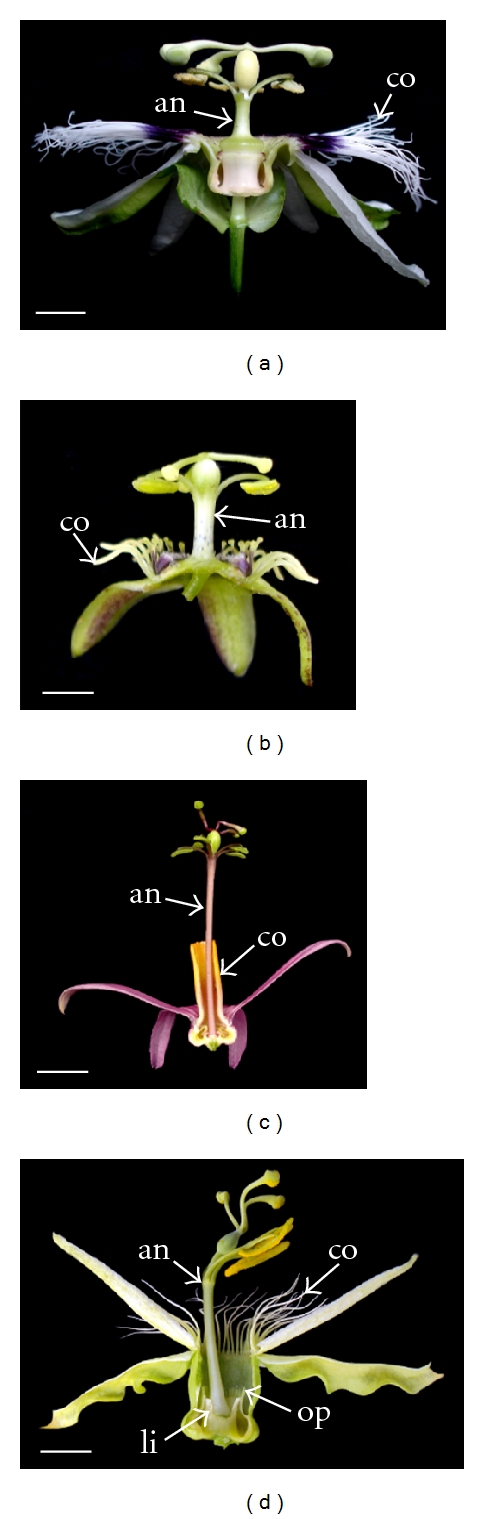
Longitudinal sections of *Passiflora* spp. flowers. (a) a large insect- (bumblebee) pollinated flower (*P. edulis*); (b) a small insect- (wasp) pollinated flower (*P. suberosa*); (c) a hummingbird-pollinated flower (*P. tulae*); (d) a bat-pollinated flower (*P. setacea*). co: corona; an: androgynophore; li: limen; op: operculum. Bars: (a), (c), and (d): 1 cm; (b): 0.2 cm.

The genus *Passiflora *comprises almost 600 species of vines, lianas, and small trees, and its diversity reaches a maximum in Central and South America [1, 2]. To the genus *Passiflora* belongs the passionfruit (*Passiflora edulis* Deg.) and other species producing ornamental flowers known collectively as “passionflowers.” Passionflowers are appreciated exactly due to a remarkable range of floral complexity and diversity. The flowers of *Passiflora *exhibits several unique floral features, including multiple series of brightly colored coronal filaments, diverse operculum morphology, an androgynophore, and elaborate floral nectary structures (Figure 1). The evolution of this extreme variation of flower morphologies is believed to be the result of interactions with a wide range of pollinators [2, 3]. Therefore, this genus is specially suited to any study on the evolution of pollination syndromes, especially those aiming to elucidate the molecular mechanisms underlying these adaptative steps.

Accordingly, one of the major challenges of current plant biology is to understand the genetic basis and molecular mechanisms of all naturally occurring developmental variation. This analysis has begun to benefit from the ever growing number of plant genomes readily available in public databases and from the availability of genomic tools aimed to identify gene functions and the mechanistic basis of phenotypes in model plant species such as *Arabidopsis thaliana*. Among these tools, expressed sequence tags (ESTs) have played significant roles in accelerating gene discovery in plants including those involved in flower development and evolution [4–7].

The main goal of our work is to understand the molecular mechanisms of the divergence of floral features among *Passiflora *species, with the aim of elucidating the role of pollinating agents in shaping the genomic shifts that lead to the actual patterns of diversification of different floral forms and structures observed in the genus. With that aim, we characterized a set of cDNA libraries from developing reproductive tissues of two divergent *Passiflora *species: *Passiflora suberosa* L. and *P. edulis *Sims. These two species were chosen due to their contrasting phenotypic characteristics: *P. edulis* produces the commercial passionfruit, with large juicy scented fruits and complete flowers while *P. suberosa* produces small fruits and small flowers lacking petals. Producing ESTs from these contrasting species might help to better study their reproductive characteristics in the future.

## 2. Material and Methods

Reproductive meristems and flower buds at different developmental stages of *P. edulis* and *P. suberosa* were collected from plants cultivated at the experimental fields at the Department of Plant Biology, IB/UNICAMP at Campinas, SP, Brazil. The samples to be used in *in situ* hybridization were fixed in 4% paraformaldehyde for 24 h at 4°C and dehydrated in an ethanol series. The samples for RNA extraction were immediately frozen in liquid nitrogen immediately after collection and stored at −80°C until use.

### 2.1. Construction of cDNA Libraries

Total RNA samples were obtained from floral buds at different developmental stages from *P. edulis *and* P. suberosa* frozen in liquid nitrogen and extracted with Trizol (Invitrogen) following the manufacturer instructions. mRNA samples were purified using Oligotex-dT (QIAGEN) resin. One to 5 *μ*g mRNA were used in cDNA synthesis and further cloning of cDNA fragments into pSPORT1 vector with the SuperScript Plasmid System for cDNA Synthesis and Cloning kit (Invitrogen), according to the manufacturer instructions. Ultracompetent *Escherichia coli *DH10B cells (Invitrogen) were electroporated (25 *μ*F; 200 Ω; 1,8 kV) with the resulting constructs, and possible transformants were plated on LB medium supplemented with ampicillin, IPTG, and X-Gal. Individual positive clones were transferred to 96-well plates containing liquid CG medium (Circle Growth, BIO 101) supplemented with. Replicates of these plates were used either for plasmid extraction and sequencing or to make glycerol stocks for library storage at −80°C.

### 2.2. Extraction of Plasmid DNA and EST Sequencing

Plasmid DNA extractions were performed according to a modified alcaline lysis method described elsewhere (http://www.protocol-online.org/prot/Protocols/Isolation-of-Plasmid-DNA-3922.html). We used the DYENAMIC kit (GE Biosciences) for sequencing reactions. Sequencing reaction products were precipitated with 95% ethanol, 1/10 reaction volume 3 M sodium acetate, and 1 g·L^−1^ glycogen. The precipitate was washed with 75% ethanol, speedvac-dryed, and loaded into a 3100 Genetic Analyser (Applied Biosystems). 

### 2.3. Bioinformatics and Sequence Analysis

We used phred [8] to determine sequence quality. The protocols for removing low-quality sequences, ribosomal sequences, and other possible contaminants as well as poly-A tails and vector sequences were described elsewhere [9]. We used CAP3 [10] for sequence clusterization. Different BLAST algorithms [11] (http://www.ncbi.nlm.nih.gov/BLAST/) were used to compare reads and the obtained clusters to publicly available sequences at the NCBI database [12]. To assemble all sequence data and their analysis, a relational database was created using in-house Pearl programming (dos Santos et al., unpublished data). This relational database allows the search of unique reads or clusters of contiguous sequences and their BLAST matches. The code used to name sequences and clusters was the same used within the frame of the SUCEST database [13]. For instance, for the sequence named PACEPE3001A01.g, “PA” designated the sequencing project, named “PASSIOMA”; “CE” referred to the sequencing lab (in this case, the Molecular and Cellular Biology Lab, CENA/USP, Brazil); “PE3” indicated the library, in this case a library made with *P. edulis* floral buds with 1 cm in length; “001” referred to plate number and “A01” to the clone position within a 96-well plate. Finally, “.g” indicated the T7 sequencing primer (alternatively, “.b” indicated that a SP6 primer was used). Clusters arbitrarily received the code of the first sequence to be included in the cluster. All sequences were automatically annotated according to their category, following the Gene Ontology Consortium (http://www.geneontology.org/) and the instructions of Telles and da Silva. [9]. Searches within the relational database can be performed using either key words or a local BLAST tool. Multiple sequence alignments of selected sequences and available putative homologs from *Arabidopsis* and/or other plant species were performed using CLUSTALX (http://www.clustal.org/). Distance trees were obtained from neighbor-joining matrices, with Bootstrap calculated from 1000 replicates and visualized with TreeView (http://taxonomy.zoology.gla.ac.uk/rod/treeview.html). Parsimony trees were obtained using hand-corrected sequence alignments with MEGA software (http://www.megasoftware.net).

### 2.4. Gene Expression Analysis

#### 2.4.1. RT-PCR

 Total RNA samples, extracted as described above were treated with DNaseI at 37°C for 15 min. Tem micrograms of total RNA were used in a Superscript II (Invitrogen) reverse transcriptase reaction with oligo (dT)_20_ following the instructions of the manufacturer. Normalized cDNA samples were used as templates in PCR reactions using gene-specific primers (Supplementary Table  1 available online at doi: 10.1155/2011/510549) and under the following conditions: 3 min of initial denaturation at 94°C; 35 cycles of 94°C for 30 s, 60°C for 45 s, 72°C for 1 min and a final extension at 72°C for 10 min. Reactions using primers for constitutive genes were used as positive controls (see Supplementary Table  1 and Supplementary Figure  1) and reactions containing RNA samples without DNAse treatment were used as negative controls. We have also tested performing the PCR for only 20 or 25 cycles (see Supplementary Figure  2). The PCR products were separated by gel electrophoresis, and the results were documented and analyzed.

### 2.5. *In Situ* Hybridization

After RT-PCR validation, the expression patterns of selected genes (Supplementary Table  1) were assessed by *in situ* hybridization. Nonradioactive probes were labeled with digoxygenin (DIG-dUTP) following the instructions of the manufacturer (Roche). The prehybridization and hybridization conditions were described elsewhere [14, 15]. Apices of reproductive shoots and flowers buds a different developmental stages were fixed and dehydrated as described above; embedded in paraffin, sectioned (8 *μ*m), and attached to glass slides previously coated with organosilane (2% solution in acetone). Prior to hybridization, the paraffin was removed from the sections by quickly washing the slides in xylol. Hybridization signal was visualized as using anti-DIG antibodies conjugated to alkaline phosphatase and a NBT/BCIP solution with levamissole (Pierce) as a substrate. Hybridized slides were observed and documented in a Zeiss Axioskop microscope.

## 3. Results and Discussion

### 3.1. Characterization of cDNA Libraries from Developing *Passiflora* Flowers

**Figure 2:**
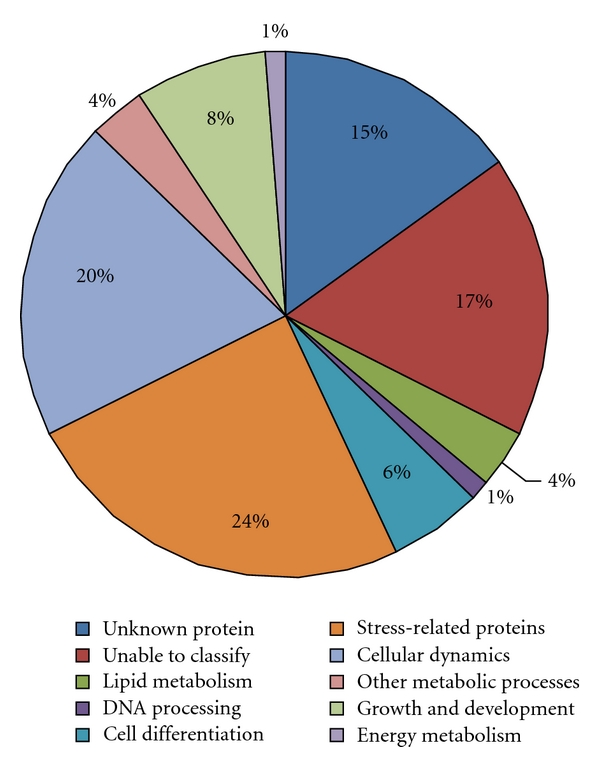
Relative frequency of *Passiflora* EST classes (all libraries combined) according to their identity and involvement in biological processes.

The data of the PASSIOMA Project can be accessed through an internet interface (passioma.ib.unicamp.br), and a login can be obtained upon contacting the authors. We produced 10,272 high-quality* Passiflora* sequences (frap/Fred >20 and >300 valid nucleotides) from 6 libraries (Table 1). All libraries contributed equally in terms of number of sequences. About half of the sequences (5,109) were from *P. Suberosa,* and the other half (5,163) were produced from *P. edulis *(Table 2). Insert amplification of 100 random clones from each library revealed inserts ranging between 500 bp and 2500 bp, with an average size of 1400 bp (Table 2). About 53% of all obtained sequences were not included in contigs and, therefore, are singletons (Table 2.). The high number of singletons reflects the low redundancy of the libraries. The *Passiflora* ESTs were annotated according to their BLAST matches and to the gene ontology (GO) [16, 17], defining functional categories to the sequences (Figure 2). The primary BLAST matches revealed three major groups of assembled *Passiflora* EST sequences with varying potential to predict their cellular function. Sequences belonging to the first group, matched sequences of known proteins with strong and nominal similarity, and are therefore likely to be transcripts of genes with similar functions (this group corresponded to 68% of all sequences). The function of the BLAST match was used to assign putative roles to this group. The second class was formed by 15% of the assembled *Passiflora* EST sequences and this group matched to “unknown protein,” “hypothetical protein,” or “putative protein,” with no indication of the function of the gene product (Figure 2). Most of the unknown proteins came from ESTs from other plant species that had been entered into the GenBank nonredundant (nr) database. The third group consisted of sequences with no matches in the GenBank nr database, and they were put into an “unable to classify” category (Figure 2); they may represent untranslated mRNAs, as well as novel *Passiflora*-specific genes. Shoemaker et al. [18] demonstrated that 13% of the soybean ESTs returned no matches after BLASTX search on trimmed sequences against the GenBank nonredundant database.

The comparison of our data with the literature describing the analysis of normalized and nonnormalized libraries of floral tissues showed similar indices of novel gene discovery [6, 7, 19–26].

As the PASSIOMA libraries were not normalized, we assumed that the most abundant transcripts found in the *Passiflora* reproductive tissues would be represented by the contig containing the largest numbers of ESTs. These contigs encode proteins generally related to stresses such as glucanases, catalases, peroxidases, jasmonate-induced proteins, and thaumatins. These results confirm previous reports that transcripts related to plant defense and stress are highly expressed during floral development [19–21]. Additionally, transcripts potentially encoding linamarases and other proteins involved in the biosynthesis of cyanogenic glycosides were generally included in clusters with up to 35 ESTs each, indicating that genes related to the biosynthesis of protecting substances are highly activated during *Passiflora* reproductive development.

**Figure 3:**
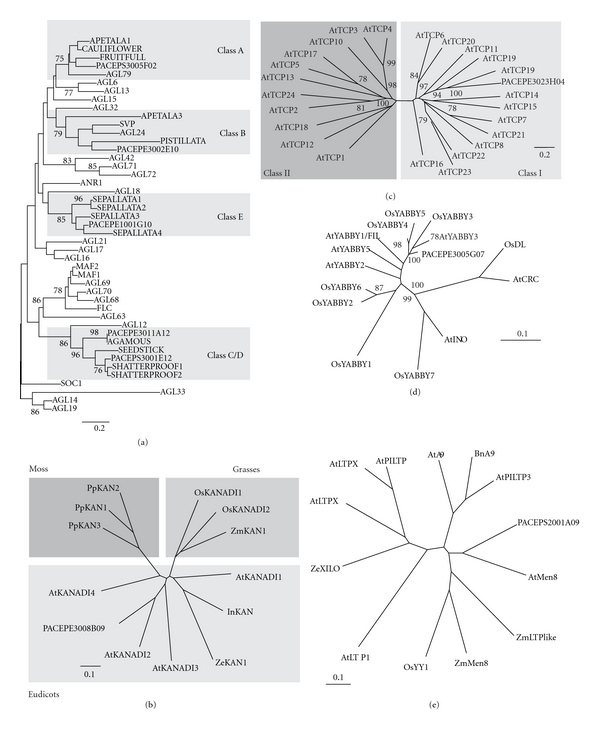
Phylogenetic analysis of selected *Passiflora* sequences. (a) sequence comparisons among *Arabidopsis* and *Passiflora* MADS-box proteins; (b) sequence comparisons among *Passiflora* KANADI protein and other plant counterparts; (c) sequence comparisons among *Arabidopsis* and *Passiflora* TCP proteins; (d) Sequence comparisons among *Arabidopsis,* rice and *Passiflora* YABBY proteins; (e) sequence comparisons among *Passiflora* Men8/9 protein and other plant counterparts. Bootstrap values above 75% are shown. Bars indicate substitution rates.

**Figure 4:**
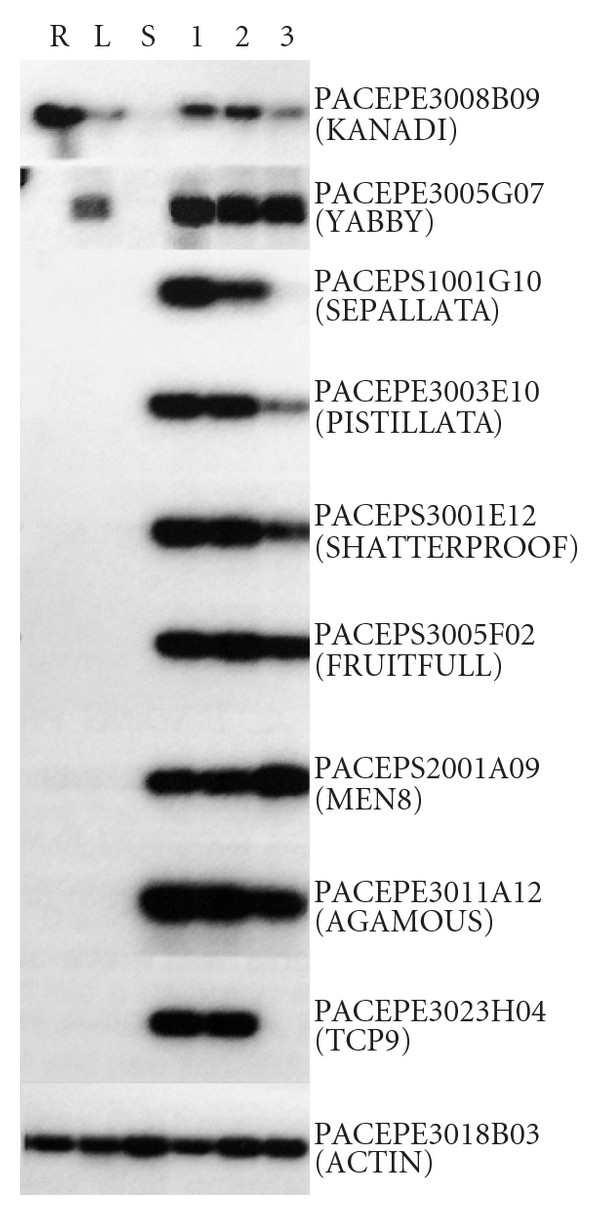
Expression patterns of *Passiflora* putative orthologs to genes involved in floral development. The *ACTIN* putative ortholog was used as a control. RT-PCR reactions were performed using as a template cDNA from roots (R), leaves (L), shoot (S), floral buds smaller than 0.5 cm (1), floral buds from 0.5 to 1.0 cm (2), and floral buds from 1.0 to 3 cm (3).

**Figure 5:**
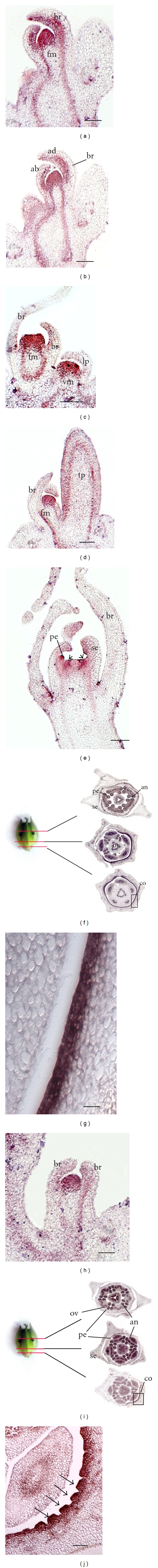
*In situ* expression patterns of putative* P. edulis* genes involved in flower development. The hybridization signal is observed as a pink-red precipitate. (a–e) and (h) show longitudinal sections though early stages of *P. edulis* flower meristems. (f, g, i, and j) show transversal sections of 4 mm long *P. edulis* flower buds. (a) the hybridization signal of PACEPE3008B09, a putative *KANADI o*rtholog, can be detected throughout the floral meristem and in the adaxial side of the bracts. As floral organ primordia are formed, the expression is confined to the adaxial side (not shown). (b) the expression of PACEPE3005G07, a putative *YABBY* ortholog, is restricted to the floral meristem dome and to the adaxial side of all floral organ primordia. (c) the hybridization signal of PACEPE3023H04, a putative *TCP9 *ortholog, is restricted to the dome of both floral and axillary vegetative meristems. (d) transcripts of PACEPE3007D03, a putative *SEPALLATA* ortholog, are detected not only in the floral meristem, but also in the tendril primordium. (e) the hybridization signal of PACEPE3002E10, a putative *PISTILLATA* ortholog, is restricted to the group of cells in whorls 3 (petal primordia) and 4 (stamen primordia, arrowheads), but later in development (f, g), it is also detected in the group of cells that originate the corona filaments (g) is a magnification of the insert shown in the lower right corner in (f). (h) transcripts of PACEPE3011A12, a putative *AGAMOUS* ortholog, are detected in the floral meristem dome early in development, but later in development (i, j), it is also detected in the group of cells that originate the corona filaments ((j) is a magnification of the insert shown in the lower right corner in (i), arrows point to primordia of the corona filaments). ab: abaxial side; ad: adaxial side; an: anther; br: bract primordium; co: corona primordia; fm: floral meristem; lp: leaf primordium; ov: ovary; pe: petal primordium; se: sepal primordium; tp: tendril primordium; vm: vegetative meristem. Bars: (a–e and h): 200 *μ*m; (g): 20 *μ*m; (j): 60 *μ*m.

**Figure 6:**
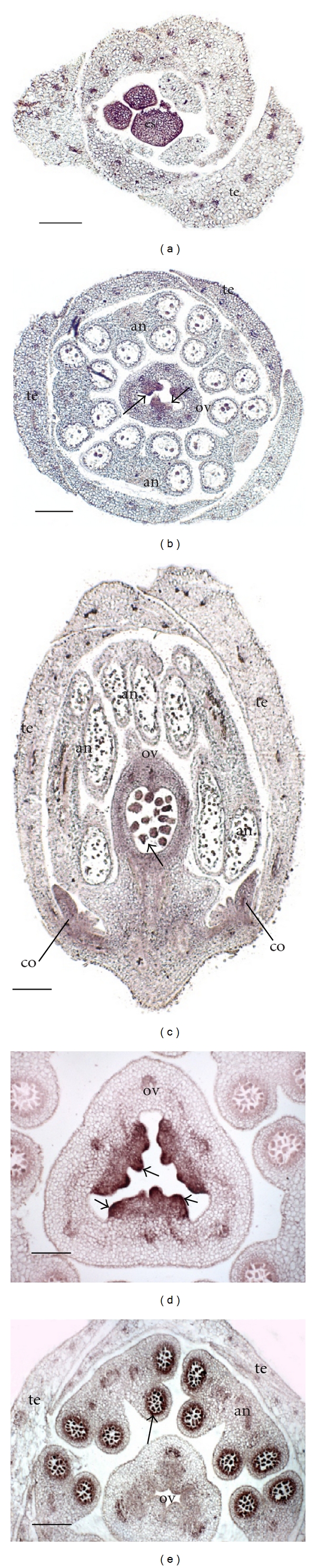
*In situ* expression patterns of putative* P. suberosa* genes involved in flower development. The hybridization signal is observed as a pink-red precipitate. (a, b, d, and e) show transversal sections though 5 mm long *P. suberosa* floral buds. (c) shows a longitudinal section. (a and b): the hybridization signal of PACEPS3005F02, a putative *FRUITFULL* ortholog, can be detected in the differentiating stigma (a) and in the ovule primordia (b, arrows). (b) is a lower section of the same floral bud shown in (a, c, and d): the expression of PACEPS3001E12, a putative *SHATTERPROOF *ortholog, is concentrated in the ovary and ovule primordia (arrow), but is also detected in the corona primordia. (d): detail of early stages of ovule primordia (arrows) showing PACEPS3001E12 expression. (e): the hybridization signal of PACEPS2001A09, a putative *MEN8* ortholog, is restricted to the tapetal cells (arrow) in developing *P. suberosa* anthers. an: anther; co: corona primordia; ov: ovary; te: tepal primordium. Bars: (a, b, and c): 200 *μ*m; (d): 80 *μ*m; (e): 150 *μ*m.

Traditionally, transcripts encoding transcription factors and homeotic genes are considered less abundant and generally are poorly represented in EST collections [6, 21–26]. However, we detected in the PASSIOMA database a number of sequences with significant similarity to transcription factors belonging to families known to play an important role during reproductive development in model species, such as the MADS-box gene family (Figure 3). Therefore, we selected some of these sequences to perform phylogenetic analyses (Figure 3) and further investigate their expression patterns using RT-PCR and *in situ* hybridization (Figures 4–6).

### 3.2. Validating *Passiflora* ESTs via Gene Expression Analysis

The efficiency of the EST approach to gene discovery in *Passiflora* could be illustrated by finding *Passiflora* EST sequences showing significant similarity to genes known to be involved in the reproductive processes in model plants such as *Arabidopsis* (Figure 3). Complementary evidences that the observed sequence similarity may reflect conservation of function might be obtained with the comparative study of their expression patterns during *Passiflora* flower development. Thus, we investigated the expression patterns of a small sample of sequences showing high-sequence similarity to known *Arabidopsis* genes, reported to be involved with key aspects of flower development.

### 3.3. RT-PCR

 Both *P. edulis* and *P. suberosa* sequences were expressed in a pattern that was very similar to their putative *Arabidopsis* orthologs, indicating a potential conservation of function. We observed two main expression patterns: sequences preferentially expressed in reproductive tissues and sequences exclusively expressed in reproductive tissues (Figure 4). Belonging to the first group are the *P. edulis* putative orthologs to the *Arabidopsis KANADI* and *YABBY *genes (Figures 3 and 4).

The products of the *Arabidopsis YABBY* and *KANADI* genes interact to establish and maintain the abaxial-adaxial polarity of all plant organs, including the reproductive ones [27, 28]. We observed the expression of the *P. edulis* putative ortholog to *KANADI* (PACEPE3008B09) in roots and leaves, besides in young floral buds (Figure 4). The transcripts of the* P. edulis* putative ortholog to *YABBY* (PACEPE3005G07) were detected in leaves and also in developing floral buds. The coexpression of PACEPE3008B09 and PACEPE3005G07 raises the possibility that the product of these sequences also interact as observed for their *Arabidopsis* counterparts.

The second class of gene expression pattern observed include those *Passiflora* sequences expressed only in floral organs. Within this class are the *Passiflora* putative orthologs to the *Arabidopsis* MADS-box genes *SEPALLATA *(*SEP*), *PISTILLATA *(*PI*), *AGAMOUS* (*AG*), *SHATTERPROOF *(*SHP*), and *FRUITFULL* (FUL).

The proteins encoded by these MADS-box genes belong to a family of transcription factors highly conserved in eukaryotes [29]. The *Arabidopsis* MADS-box family has 107 members, indicating the relevance of this transcription factor-encoding genes to plants [30]. The biological function of most genes of the family is unknown, but three classes of MADS-box genes (named A, B, and C) are responsible to the establishment of the identities of all floral organs according to the classical ABC Model of flower development proposed by [31]. According to this model, the expression of A class MADS-box gene *APETALA1* (*AP1*) in the periphery of the floral meristem determines the formation of sepals; the combined expression of *AP1* and B class genes *APETALA3* (*AP3*) and *PISTILLATA* (*PI*) in a ring of cells corresponding to the second whorl determines the differentiation of petals; the coexpression of *PI, AP3,* and C class gene *AGAMOUS* (*AG*) in the third whorl establishes the formation of stamens and, finally, the expression of *AG* alone in the center of the floral meristem determines the differentiation of carpels [31]. Later on, four E class paralogous genes *SEPALLATA *were added to the model, where they determine the “floral” identity of all ABC-expressing organs [32, 33]. All the ABCE genes are expressed exclusively in floral tissues in *Arabidopsis*. Accordingly, their *Passiflora* putative orthologs (Figure 3) were also expressed only during flower development (Figure 4).

The* Arabidopsis SHATTERPROOF *(*SHP*) and *FRUITFULL* (*FUL*) genes also belong to the MADS-box gene family, and they are involved in *Arabidopsis* carpel and fruit development [34–36]. The transcripts of their *Passiflora* putative orthologs were found only during floral development (Figure 4). This expression pattern was also observed for orthologs of these genes in other plant species [34–37].

 The homologs of *MEN8* from *Arabidopsis* and *Brassica oleracea* are expressed exclusively in floral tissues, more specifically during anther development [38]. The *MEN8* gene encodes a lipid transfer protein, apparently involved with the deposition of pollenkit on the surface of developing pollen grains [38]. In agreement to this putative function, the *Passiflora MEN8* putative ortholog (PACEPS2001A09, see Figure 3) was also expressed only in floral tissues (Figure 4).

 There are 24 members in the *Arabidopsis* TCP family of transcription factors, organized in two subfamilies [39]. The members of subfamily I, which include *TCP9*, generally show flower-specific expression, even in monocots such as rice [40]. The *TCP* genes are involved with the maintenance of cellular proliferation within the floral meristem and with plant organ growth. Therefore, *tcp* mutants frequently are affected in the size and shape of floral organs [39, 41]. According to its possible role during early floral development, the transcripts of the putative *Passiflora* ortholog of *TCP9* (Figure 3) were only detected during early flower development (Figure 4).

### 3.4. *In Situ* Hybridization

In order to corroborate and complement the RT-PCR results, we performed *in situ* hybridization experiments. The presence of transcripts was investigated in histological sections of reproductive apices and floral buds of *P. edulis* (Figure 5) and *P. suberosa* (Figure 6) at different developmental stages.

The RT-PCR results for the *P. edulis* putative ortholog of *KAN *(PACEPE3008B09) indicated that it is predominantly expressed in reproductive tissues, but, in conformity to the predicted function of the *Arabidopsis* KAN, it was also expressed in vegetative tissues. The hybridization signal of PACEPE3008B09 was detected throughout the* P. edulis* floral meristem and in the adaxial side of the bracts (Figure 5(a)). Its expression was consistently conserved in the adaxial side of other floral organ primordia during their early developmental stages (data not shown). *Arabidopsis* mutants to two of the *KAN* paralogous genes show a substitution of abaxial cell types to adaxial ones and the ectopic expression of *KAN* in leaf or floral organ primordia results in the abaxialization of the tissues as well as defects in vascular differentiation and abnormalities on the expansion of laminar organs such as sepals and petals [28]. These phenotypes suggest that the KAN genes perform an important role in the determination of adaxial-abaxial organ polarity.

The asymmetric distribution of *KAN* transcripts in organ primordia is related to the differential activation of PHANTASTICA (PHAN), PHABULOSA (PHAB), PHAVOLUTA (PHAV) as well as the *YABBY* genes [27, 28]. We did not detect any sequences in the PASSIOMA database showing significant similarity to PHAN, PHAB, or PHAV, but we found the sequence PACEPE3005G07, which shows high similarity to the *Arabidopsis YABBY1/FILAMENTOUS FLOWER *(*YAB1/FIL*). The *Arabidopsis* YABBY family of transcription factors has 6 members differentially expressed during plant development [27, 42]. The current models predict that the YABBY transcription factors promote the identity of abaxial cells in all plant lateral organs, in association with the *KANADI* gene products [27, 28]. In *Arabidopsis*, *YAB1/FIL* is expressed in the embryonic root and in all shoot meristem products after germination. In the aerial organs, its expression is restricted to the adaxial side [42, 43], thus co-expressing with *KAN*. Accordingly, PACEPE3005G07 transcripts were detected in the same floral tissues as the putative ortholog of *KAN *(PACEPE3008B09). PACEPE3005G07 hybridization signal was detected uniformly in the shoot apical meristem as well as in the early tendril primordia and in the adaxial side of leaf primordia (data not shown). During early floral meristems development, PACEPE3005G07 transcripts were detected in the adaxial side of floral organ primordia (Figure 5(b)). Further investigation is needed in order to clarify if the products of the co-expressing putative *KAN *and *YAB Passiflora* orthologs interact, as their *Arabidopsis* counterparts do.

The *Arabidopsis* TCP family of transcription factors has 24 members organized into two subfamilies [39, 41]. *TCP9* belongs to subfamily I. The members of this subfamily are specifically expressed in lateral meristems and floral tissues and are involved in the maintenance of cell proliferation, generally associated to the control of size and shape of floral organs [39, 41]. The expression of the *Passiflora* putative ortholog of *TCP9*, PACEPE3023H04, agreeing with its expected function in controlling cell proliferation, is concentrated both in the vegetative lateral meristem and floral meristems (Figure 5(c)). The members of the TCP family are highly conserved among angiosperms and one of the maize homologs, TEOSINTHE BRANCHED1, is important to the determination of plant architecture in modern corn cultivars [44]. Future experiments involving transgenic *P. edulis* plants, in which PACEPE3023H04 expression is modulated, may shed light into his function during *Passiflora* development.

The *Arabidopsis SEPALLATA* (*SEP*) genes act redundantly to specify all floral whorls, in combination with the ABC-class MADS-box genes [45]. Our *in situ* hybridization results indicated that PACEPE1001G10, the putative *P. edulis* ortholog of *SEP3* was expressed in the developing tendril primordia as well as during early flower meristem development, being excluded from the floral bract primordia (Figure 5(d)). PACEPE1001G10 transcripts were not detected in the shoot apical meristem or in the lateral vegetative meristems (data not shown). The expression of a putative *Passiflora* ortholog of *SEP* in tendril primordia is somewhat surprising, but Carmona et al. [46] reported the expression of floral MADS-box genes in developing grape tendril primordia, suggesting that grape tendrils are modified flowers. Interestingly, it was recently suggested that *Passiflora* tendrils might be modified flowers as well [47]. Additionally, *SEP* orthologs in other plant species seem to have acquired diverse roles in inflorescence architecture specification and during fruit maturation [48].

The transcripts of PACEPE3002E10, the putative *P. edulis* ortholog of the *Arabidopsis PISTILLATA* gene, were detected in the developing floral meristem, in cellular domains corresponding to the forming petal and sepal whorls, even before the corresponding primordia were formed (Figure 5(e)). The presence of PACEPE3002E10 transcripts in the second and third whorls of floral organs was maintained until late during flower bud development (Figure 5(f)). Accordingly, the *Arabidopsis* B class MADS-box genes *AP3* and *PI* are expressed early during floral meristem development in a ring of cells that will give rise to petal and stamen primordia [49]. Interestingly, PACEPE3002E10 transcripts were also detected in the group of cells from which the corona filaments will develop (Figures 5(f) and 5(g)), raising the possibility that B class MADS-box genes might be involved in the determination of corona identity.

In the classical ABC model [31], *AG *is the only *Arabidopsis* C class gene. Its expression is important to the determination of stamen (when coexpressed with B class genes) and carpel identities (when expressed in the center of the floral meristem). Generally, the mutations in the *AG* locus promote the production of indeterminate flowers made of successive whorls of sepals and petals [31]. The *Arabidopsis AG* expression is restricted to the floral meristematic dome and in posterior stages, *AG *transcripts can be found only in the two innermost floral whorls [50, 51]. In agreement with its putative role as a potential *AG* ortholog, PACEPE3011A12 transcripts were detected in the central region of early floral meristems (Figure 5(h)) and later found in developing stamen and carpel primordia (Figure 5(i)). Additionally, PACEPE3002E10 transcripts were also detected in the developing primordia of corona filaments (Figures 5(i) and 5(j)), indicating a possible coexpression with the B class putative ortholog PACEPE3002E10, suggesting that a combination of B and C classes MADS-box genes might be involved in corona development. Further research is necessary to confirm this possibility.

During *Arabidopsis* flower development, *FUL* transcripts are detected in the early stages of carpel development. *FUL* transcripts continue to be detected throughout carpel development and later accumulate in ovule primordia [35, 52, 53]. The transcripts of PACEPS3005F02, the putative *P. suberosa* ortholog of *FUL*, were detected in *P. suberosa* carpel tissues upon their differentiation (data not show). Later in development, hybridization signal was detected in differentiating stigmatic tissues (Figure 6(a)) and in the ovary, where it was concentrated in the ovule primordia (Figure 6(b)). Among the proposed roles for the *Arabidopsis FUL* gene product is the coordination of cell-cell interactions that leads to the differentiation of a dehiscence zone in the mature fruit [36]. Since *P. suberosa* fruits are indehiscent, a detailed analysis of PACEPS3005F02 expression patterns is necessary to suggest a putative function for this gene in *Passiflora* reproductive development. Nevertheless, putative orthologs of *FUL *and *SHP* were described to be expressed during fruit development of peach (*Prunus persica*) [37]. Similar to *Passiflora*, peach also has indehiscent fleshy fruits, and, in this species, it was suggested that these MADS-box genes might be related to specific peach fruit features such as the differentiation of the split junction between the nut and the fruit flesh, an important characteristic to the industrial processing of peach fruits [37]. Therefore, there might be interesting features associated to the function of the PACEPS3005F02 product, which may be of relevant interest to the passionfruit juice industry. This gene product is of special interest, since its interaction partner in *Arabidopsis, SHP*, also has a potential ortholog in *P. suberosa*, PACEPS3001E12.

 PACEPS3001E12 expression was detected early during *P. suberosa* carpel development (data not show). Later in development, PACEPS3001E12 transcripts were detected in the ovary tissues, including the ovary wall and ovule primordia (Figures 6(c) and 6(d)). Additionally, a hybridization signal was detected in the developing corona filaments (Figure 6(c)). The expression in the corona filaments is intriguing, and further analysis will be necessary to uncover the role of PACEPS3001E12 transcripts during corona development. Generally, only one ortholog of the *Arabidopsis* paralogous genes *SHATTERPROOF 1* and *2* (*SHP1* and *SHP2*) is found in angiosperms [37]. Therefore, it is probable that the *Arabidopsis SHP1*/*SHP2 *duplication is recent and is present only within Brassicaceae [36]. In *Arabidopsis*, *SHP1*/*SHP2 *are widely expressed in the medial tissues of the gynoecium, including the replum, the valve margins, septum and ovule primordia. Later in development, their expression is excluded from the replum tissues, where *SHP1*/*SHP2 *are downregulated by FUL [36].

## 4. Conclusions and Perspectives

The sequencing and characterization of *Passiflora* ESTs can contribute to establish a consistent genomic basis for the analysis of the molecular regulation of floral organ development. The use of this approach was very fruitful in revealing sequences potentially involved in different aspects of floral development such as the determination of floral organ identity (with the characterization of putative *Passiflora* orthologs of MADS-box genes), floral organ symmetry (with the characterization of putative *Passiflora* orthologs of *KAN* and *YAB* genes) and the differentiation of specialized tissues within a given organ (with the characterization of a putative *Passiflora* ortholog of* MEN8*). Additionally, the EST sequences of the PASSIOMA database might provide useful resources for the development of EST-based markers for important agronomic traits as well as to the establishment of genomic tools to study the naturally occurring floral diversity among *Passiflora *species. The manipulation of the expression patterns of these candidate genes in transgenic *Passiflora* plants sounds as an obvious continuation of our studies and experiments with this aim are underway in our group.

## Supplementary Material

Supplementary Figure 1: describes RT-PCR reactions performed using primers for constitutive genes such as 25S and GADPH.
Supplementary Figure 2: describes RT-PCR reactions performed using primers for the PePISTILLATA gene.
Supplementary Table 1: contains primer sequences used for RT-PCR.
Supplementary Table 2: contains the list of accession numbers for the sequences used in the phylogenetic analyses.

## Figures and Tables

**Table 1:** The cDNA libraries obtained from* Passiflora* reproductive tissues.

Library	Source	*Passiflora* species
PE1	Apices (about 1 cm) from adult plants at the reproductive state	*P. edulis*
PE2	Developing flower buds (0.2 to 0.5 cm)	*P. edulis*
PE3	Developing flower buds (0.5 to 1 cm)	*P. edulis*
PS1	Apices (about 1 cm) from adult plants at the reproductive state	*P. suberosa*
PS2	Developing flower buds (0.1 to 0.5 cm)	*P. suberosa*
PS3	Developing flower buds (0.5 cm to preanthesis)	*P. suberosa*

**Table 2:** Characterization of the cDNA libraries obtained from* Passiflora* reproductive tissues at different developmental stages.

	*P. edulis*	*P. suberosa*
Number of valid ESTs	5,163	5,109
Clusters	597	588
Singletons	2,731	2,743
Cluster composition^ a^	3	2
Mean insert size (bp)	1,386	1,423
Mean ORF size (bp)	482	463
Novelty (%)^ b^	86.45	89.65

^a^: Mean number of ESTs in each cluster.

^b^: Mean proportion of a novel singleton or cluster formed for each new sequence introduced in the database (considering all libraries).
