# Supplementary material for: PASSIOMA: Exploring Expressed Sequence Tags during Flower Development in Passiflora spp
Source: Comp Funct Genomics. 2012 Mar 22;2012:510549. doi: 10.1155/2012/510549 (PMC3318222; doi:10.1155/2012/510549)

Supplementary Figure 1: RT-PCR reactions performed using primers for constitutive genes such as 25S and GADPH and cDNA from roots (R), leaves (L), shoot (S), floral buds smaller than 0.5 cm (1), floral buds from 0.5 to 1.0cm (2) and floral buds from 1.0 to 3cm (3) as templates.

Supplementary Figure 2: RT-PCR reactions performed using primers for the PePISTILLATA gene and cDNA from roots (R), leaves (L), shoot (S), floral buds smaller than 0.5 cm (1), floral buds from 0.5 to 1.0cm (2) and floral buds from 1.0 to 3cm (3) as templates. Three different cycle numbers were tested. Note differences in band intensity were proportional.

Supplementary Table 1

Primer sequences used for RT-PCR

PRIMER	Gene (clone)	Primer sequence	Fragment size
**PSEPF**<br>**PSEPR**	SEPALLATA<br>(PACEPE1001G10)	5´-GATGAGGAGGCATCAAATGT-3´<br>5´-CTCAGGAGAGCTTTCAAGGA-3´	561bp
**PPISF**<br>**PPISR**	PISTILLATA<br>(PACEPE3002E10)	5´-GTCTGCTCAAGAAAGCCTAT-3´<br>5´-CTGTTCTCTGTGATGCTGAG-3´	425bp
**PAGAF**<br>**PAGAR**	AGAMOUS<br>(PACEPE3011A12)	5´-CGCCGAAATCGAGTACATGC-3´<br>5´-CTTGCTTGAGGTGCTATCTT-3´	684bp
**PFULF**<br>**PFULR**	FRUITFULL<br>(PACEPS3005F02)	5´-GGCAATGGCTGAGCAGGAAG-3´<br>5´-GTTTATTAGGCCAGGCCCAT-3´	463bp
**PSHTF**<br>**PSHTR**	SHATTERPROOF<br>(PACEPS3001E12)	5´-GTGATGTTCACCTGAGAACA-3´<br>5´-CATCGGTTCACCTTCCTCTT-3´	608bp
**PAMEN8F**<br>**PAMEN8R**	MEN8<br>(PACEPS2001A09)	5´-CGCGTCCGTGATTGAACTGC-3´<br>5´-CTGACGCTCCTGAGTGATCA-3´	532bp
**PKANF**<br>**PKANR**	KANADY<br>(PACEPE3008B09)	5´-GCAGTACGGTCGGAATTCCC-3´<br>5´-GTCGATTACGCGTCCGTGAT-3´	486bp
**PYABF**<br>**PYABR**	YABBY<br>(PACEPE3005G07)	5´-GAACTGCAATCTTTCACCT-3´<br>5´-CTGTGTCTGAGGCTAATGC-3´	654bp
**PTCPF**<br>**PTCPR**	TCP9<br>(PACEPE3023H04)	5´-CTGACGCTCCTGAGTGATCA-3´<br>5´-GCTCAGTTCTATCAGCAAGA-3´	620bp
**PACTINF**<br>**PACTINR**	Actin*<br>(PACEPE3018B03)	5´-ACCCACAACAGAGCACAACA-3´<br>5´-CCAGTGGTGGTGAAGGAGTAA-3´	658bp
**P25SRRNAF**<br>**P25SRRNAR**	25S*<br>(PACEPE3028D02)	5´-CCTTCGCGCTCTCTGTGAT-3´<br>5´-GCTGTCAAACTGGGCAAGAT-3´	404bp
**PGADPHF**<br>**PGADPHR**	GADPH*<br>(PACEPE3001F02)	5´-AGCCAAACCATCTTGTCAGG-3´<br>5´-GAAGAGCAAGGCAGTGTGGT-3´	643bp

*** These genes were used as positive controls, as they are supposed to express constitutively.**

Supplementary Table 2

List of accession numbers for the sequences used in the phylogenetic analyses

**Gene**	**Accession number**
A. thaliana MADS (APETALA1)	At1g69120
A. thaliana MADS (CAULIFLOWER)	At1g26310
A. thaliana MADS (FRUITFULL)	At5g60910
A. thaliana MADS (AGL79)	At3g30260
A. thaliana MADS (AGL6)	At2g45650
A. thaliana MADS (AGL13)	At3g61120
A. thaliana MADS (AGL15)	At5g13790
A. thaliana MADS (AGL32)	At5g23260
A. thaliana MADS (APETALA3)	At3g54340
A. thaliana MADS (SVP)	At2g22540
A. thaliana MADS (AGL24)	At4g24540
A. thaliana MADS (PISTILLATA)	At5g20240
A. thaliana MADS (AGL42)	At5g62165
A. thaliana MADS (AGL71)	At5g51870
A. thaliana MADS (AGL72)	At5g51860
A. thaliana MADS (ANR1)	At2g14210
A. thaliana MADS (AGL18)	At3g57390
A. thaliana MADS (SEPALLATA1)	At5g15800
A. thaliana MADS (SEPALLATA2)	At3g02310
A. thaliana MADS (SEPALLATA3)	At1g24260
A. thaliana MADS (SEPALLATA4)	At2g03710

Supplementary Table 2 (cont.)

Gene	Accession number
A. thaliana MADS (AGL21)	At4g37940
A. thaliana MADS (AGL17)	At2g22630
A. thaliana MADS (AGL16)	At3g57230
A. thaliana MADS (MAF2)	At5g65050
A. thaliana MADS (MAF1)	At1g77080
A. thaliana MADS (AGL69)	At5g65070
A. thaliana MADS (AGL70)	At5g65060
A. thaliana MADS (AGL68)	At5g65080
A. thaliana MADS (FLC)	At5g10140
A. thaliana MADS (AGL63)	At1g31140
A. thaliana MADS (AGL12)	At1g71692
A. thaliana MADS (AGAMOUS)	At4g18960
A. thaliana MADS (SEEDSTICK)	At4g09960
A. thaliana MADS (SHATTERPROOF1)	At3g58780
A. thaliana MADS (SHATTERPROOF2)	At2g42830
A. thaliana MADS (SOC1)	At2g45660
A. thaliana MADS (AGL33)	At2g26320
A. thaliana MADS (AGL14)	At4g11880
A. thaliana MADS (AGL19)	At4g22950
A. thaliana TCP1	At1g67260
A. thaliana TCP2	At4g18390
A. thaliana TCP3	At1g53230

Supplementary Table 2 (cont.)

**Gene**	**Accession number**
A. thaliana TCP4	At3g15030
A. thaliana TCP5	At5g60970
A. thaliana TCP6	At5g41030
A. thaliana TCP7	At5g23280
A. thaliana TCP8	At1g58100
A. thaliana TCP9	At2g45680
A. thaliana TCP10	At2g31070
A. thaliana TCP11	At2g37000
A. thaliana TCP12	At1g68800
A. thaliana TCP13	At3g02150
A. thaliana TCP14	At3g47620
A. thaliana TCP15	At1g69690
A. thaliana TCP16	At3g45150
A. thaliana TCP17	At5g08070
A. thaliana TCP18	At3g18550
A. thaliana TCP19	At5g51910
A. thaliana TCP20	At3g27010
A. thaliana TCP21	At5g08330
A. thaliana TCP22	At1g72010
A. thaliana TCP23	At1g35560
A. thaliana TCP24	At1g30210
A. thaliana KANADI1	At5g16560
A. thaliana KANADI2	At1g32240

Supplementary Table 2 (cont.)

**Gene**	**Accession number**
A. thaliana KANADI3	At4g17695
A. thaliana KANADI4	At5g42630
A. thaliana LTP1	At2g38540
A. thaliana MEN8	At5g52160
A. thaliana A9	At5g62080
A. thaliana LTPx	At2g13820
A. thaliana PILTP	At5g64080
A. thaliana PILTP3	At5g07230
A. thaliana YABBY1/FIL	AF136538
A. thaliana YABBY2	AF136539
A. thaliana YABBY3	AF136540
A. thaliana YABBY4/INO	AF136547
A. thaliana YABBY5	AK119091
A. thaliana YABBY6/CRC	AF132606
Brassica napus A9	CAA43890
Ipomoeaea nil KANADI	Q2PF88
Oryza sativa KANADI1	Os09g23200
Oryza sativa KANADI2	Os08g33050
Oryza sativa YABBY1	AB274013
Oryza sativa YABBY2	AB274014
Oryza sativa YABBY3	AB274015
Oryza sativa YABBY4	AB274016
Oryza sativa YABBY5	AB274017

Supplementary Table 2 (cont.)

**Gene**	**Accession number**
Oryza sativa YABBY6	AB274018
Oryza sativa YABBY7	AB274019
Oryza sativa DL	AB106553
Oryza sativa YY1	BAA23617
Physcomytrella patens KANADI1	EDQ81763
Physcomytrella patens KANADI2	EDQ73668
Physcomytrella patens KANADI3	EDQ65761
Zinia elegans KANADI1	BAD11206
Zinia elegans XILO	BAD24657
Zea maysLTP-like	CAB65538
Zea mays MEN8	CAA11913
Zea mays KANADI1	A0S5W2

Supplementary Figure 1

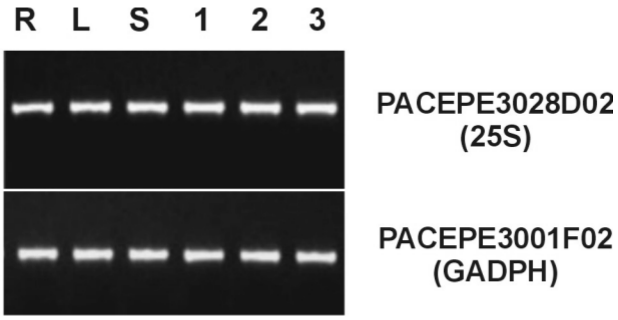

Supplementary Figure 2

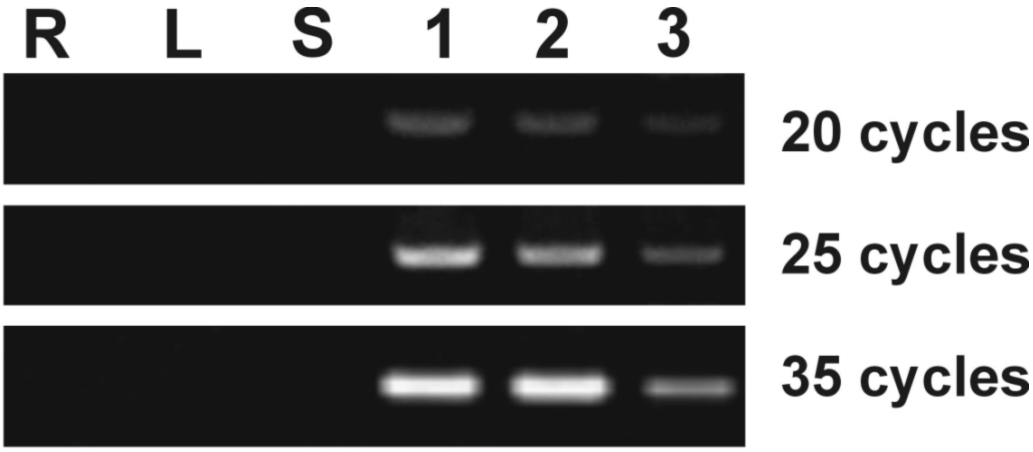

Supplement: Supplementary file 1 — Supplementary Figure 1: describes RT-PCR reactions performed using primers for constitutive genes such as 25S and GADPH. Supplementary Figure 2: describes RT-PCR reactions performed using primers for the PePISTILLATA gene. Supplementary Table 1: contains primer sequences used for RT-PCR. Supplementary Table 2: contains the list of accession numbers for the sequences used in the phylogenetic analyses. [file 510549.f1.pdf]
